# Cardiovascular Health in Anxiety or Mood Problems Study (CHAMPS): study protocol for a randomized controlled trial

**DOI:** 10.1186/s13063-015-1109-z

**Published:** 2016-01-11

**Authors:** Phillip J. Tully, Deborah A. Turnbull, John D. Horowitz, John F. Beltrame, Terina Selkow, Bernhard T. Baune, Elizabeth Markwick, Shannon Sauer-Zavala, Harald Baumeister, Suzanne Cosh, Gary A. Wittert

**Affiliations:** Department of Rehabilitation Psychology and Psychotherapy, Institute of Psychology, University of Freiburg, Engelbergerstr. 41, Freiburg, 79085 Germany; INSERM, U897-Epidemiology and Biostatistics, Bordeaux, France; School of Psychology, The University of Adelaide, Adelaide, Australia; Department of Cardiology, Basil Hetzel Institute, The Queen Elizabeth Hospital and The University of Adelaide, Adelaide, Australia; Discipline of Psychiatry, The University of Adelaide, Adelaide, Australia; Department of Psychiatry, The Queen Elizabeth Hospital, Woodville West, Australia; Center for Anxiety & Related Disorders, Department of Psychology, Boston University, Boston, USA; Freemasons Foundation Centre for Men’s Health, Discipline of Medicine, School of Medicine, The University of Adelaide, Adelaide, Australia

**Keywords:** Depression, major depression, anxiety, anxiety disorders, cardiovascular disease, coronary heart disease, randomized controlled trial, cognitive behavioral therapy, diagnosis, prognosis

## Abstract

**Background:**

Previous psychological and pharmacological interventions have primarily focused on depression disorders in populations with cardiovascular diseases (CVDs) and the efficacy of anxiety disorder interventions is only more recently being explored. Transdiagnostic interventions address common emotional processes and the full range of anxiety and depression disorders often observed in populations with CVDs. The aim of CHAMPS is to evaluate the feasibility of a unified protocol (UP) for the transdiagnostic treatment of emotional disorders intervention in patients recently hospitalized for CVDs. The current study reports the protocol of a feasibility randomized controlled trial to inform a future trial.

**Methods/Design:**

This is a feasibility randomized, controlled trial with a single-center design. A total of 50 participants will be block-randomized to either a UP intervention or enhanced usual care. Both groups will receive standard CVD care. The UP intervention consists of 1) enhancing motivation, readiness for change, and treatment engagement; (2) psychoeducation about emotions; (3) increasing present focused emotion awareness; (4) increasing cognitive flexibility; (5) identifying and preventing patterns of emotion avoidance and maladaptive emotion-driven behaviors (EDBs, including tobacco smoking, and alcohol use); (6) increasing tolerance of emotion-related physical sensations; (7) interoceptive and situation-based emotion-focused exposure; and (8) relapse prevention strategies. Treatment duration is 12 to 18 weeks. Relevant outcomes include the standard deviation of self-rated anxiety, depression and quality of life symptoms. Other outcomes include intervention acceptability, satisfaction with care, rates of EDBs, patient adherence, physical activity, cardiac and psychiatric readmissions. Parallel to the main trial, a nonrandomized comparator cohort will be recruited comprising 150 persons scoring below the predetermined depression and anxiety severity thresholds.

**Discussion:**

CHAMPS is designed to evaluate the UP for the transdiagnostic treatment of emotional disorders targeting emotional disorder processes in a CVD population. The design will provide preliminary evidence of feasibility, attrition, and satisfaction with treatment to design a definitive trial. If the trial is feasible, it opens up the possibility for interventions to target broader emotional processes in the precarious population with CVD and emotional distress.

**Trial registration:**

ACTRN12615000555550, registered on 29/05/2015

## Background

Depression disorders are severely disabling and common in cardiovascular disease (CVD) populations and portend poorer cardiovascular outcome [1, 2] and high costs [3]. Consequently, the treatment of depression disorders has dominated the contemporary psychological intervention landscape in populations with CVD for the past 25 years. Considering that depression is a putative CVD risk factor that is also modifiable, a common hypothesis is that psychosocial interventions in this population would lead to significant benefits to an otherwise poorer cardiovascular prognosis. However, prior intervention efforts addressing depressive symptoms among CVD patients have not typically produced a sizeable clinical impact upon either depression or major CVD morbidity [4], and these unsatisfying findings raise the question of how existing interventions could be improved.

Several lines of evidence indicate that interventions focusing solely on depressive symptoms in CVD patients may be too narrow in focus. Specifically, an accumulating body of work shows that anxiety disorders confer CVD morbidity risk independent of or in conjunction with depression [5–14]. Indeed, the clinical reality of comorbid depression and anxiety that is evident in CVD populations [15] closely parallels findings in clinical and epidemiological samples [16, 17]. Nonetheless, the heightened CVD risk is not constrained to simply depression and anxiety disorders given that positive associations between CVD events have been reported in relation to hostility, anger, stress, social isolation, worry, rumination, somatic depressive symptoms, anxiety sensitivity, phobic anxiety and the specific combination of negative affectivity and social inhibition [15, 18–22]. Collectively these findings point to the likelihood that common processes underlying negative emotions generally also portend CVD risk [19], raising the possibility that an intervention that transcends diagnostic boundaries and targets core emotional processes would be a step toward improving mental health interventions among CVD populations.

### Limitations of the extant depression intervention findings in CVD

Several lines of evidence raise the possibility that interventions focusing solely on depression disorders have been ineffective in the population with CVDs. First, prior controlled efforts to address depression in patients with coronary artery disease [4, 23] and heart failure [24] via psychological and pharmacological interventions have reported only small, albeit significant, effects on depression symptoms. Strikingly, the effect sizes for depression symptom reduction are markedly smaller in CVD samples than that observed in other chronic diseases such as Type II diabetes samples [25]. Collective findings have prompted a closer examination of the specific therapeutic components of interventions for depression used in CVD samples that might lead to an improved outcome [26], as well as an exploration of novel methods of mental health service delivery such as collaborative care [27]. Intriguingly, neither avenue of empirical enquiry has consistently yielded moderate or larger effect sizes for depression symptom reduction to date. Likewise, despite initially promising findings from one collaborative care randomized controlled trial (RCT) suggesting a reduction in major adverse cardiac events (MACE) [28], these findings were not sustained in the longer term [23, 29]. Together, these findings point to the strong possibility that mental health interventions exclusively targeting depression in CVDs are incomplete and too narrow in focus.

### Anxiety burden and effects on CVD prognosis

Parallel to what has been reported for depression, an emerging literature has uncovered an association between anxiety with CVD morbidity [8, 15], prompting questions regarding whether depression is a discrete psychiatric risk factor for MACE [11, 15]. Specifically, two recent meta-analyses indicated that post-traumatic stress disorder was associated with a twofold recurrent acute coronary syndrome risk [7] whereas generalized anxiety disorder (GAD) was associated with a 21 % increase in MACE risk [15]. Although the association between panic disorder and CVD events is tenuous [9, 30–34], panic disorder nonetheless signifies high emergency department utilization for chest pain and diagnostic tests to rule out acute coronary syndromes [35], representing a major burden on the healthcare system.

### Importance of anxiety intervention in CVDs

Alongside depression, the American Heart Association [36] and the German Cardiac Society [21] recommend comprehensive assessment of anxiety disorders in CVD patients. In depressed CVD patients, the prevalence of comorbid anxiety disorders is 30 % to 50 % [15, 27]. In addition, 50 % of cardiac patients presenting for psychotherapy meet at least one anxiety disorder diagnosis [37, 38], and higher anxiety is associated with depression treatment resistance [39]. Despite the prevalence of anxiety disorders in CVDs exceeding community estimates [17] and comparable to depression prevalence in CVD [15], no clinical guidelines exist for treatment when anxiety disorder comorbidity is present in this population [40]. Moreover, with increasing recognition of the necessity to formulate interventions matching the complexity of CVD populations [37, 41] and the requirement for mental health interventions to match transdiagnostic developments in clinical psychology [42–47], the use of depression disorder-only treatments may become increasingly obsolete.

### Previous work supporting the need for a transdiagnostic approach in CVDs

Our recent work supports the assertion that an expansion of mental health interventions beyond depression to incorporate comorbid anxiety disorders is warranted in populations with CVDs. A screen-and-treat model of care for heart failure patients was undertaken in three South Australian tertiary hospitals where we observed that panic disorder and anxiety due to a medical condition was associated with high emergency department utilization [48]. Furthermore, a reduction in CVD readmissions was observed with CBT for GAD [49]. We hypothesized that an intervention focusing on somatic anxiety symptoms led to a significant reduction in CVD hospital readmissions and to an improved depression response [49].

Despite strong evidence collectively demonstrating how anxiety disorders are common and necessitate treatment in CVDs, a critical absence of interventions with broader reach beyond depression exists, which forms a major oversight in improving upon existing mental health interventions. Therefore, treatment of a broader range of emotional disorders and their common emotional processes likely would be an innovative step toward improving mental health interventions and cardiovascular health among CVD populations. Targeting the most common psychological processes that confer CVD risk with a single set of therapeutic principles would open the possibility for a more parsimonious application of effective mental health interventions to a broader range of CVD patients. Herein, we outline the methodology for a study of this type based on the unified protocol (UP) for transdiagnostic treatment of emotional disorders [42, 44–46].

### Study objectives

The aim of the Cardiovascular Health in Anxiety and Mood Problems Study (CHAMPS) is to prospectively study the UP in patients with a recent CVD hospitalization and comorbid depression and/or anxiety. The outcomes of interest include the standard deviation of self-rated depression, anxiety and quality of life symptoms. Other relevant outcomes include rates of EDBs (tobacco smoking and alcohol use), physical activity levels, patient adherence, cardiac and psychiatric readmissions. A third objective is to explore trajectories of emotional distress in relation to EDBs and psycho-behavioral CVD risk factors. The data obtained will provide an estimate of effect size for a larger more definitive trial.

## Methods

### Study design

**Fig 1:**
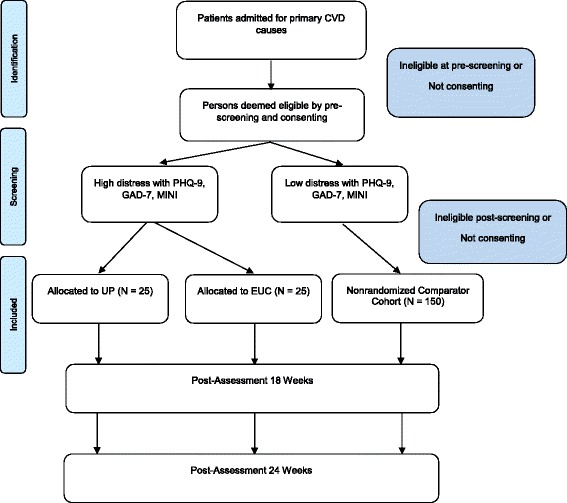
Flow chart of CHAMPS participants through the study. CVD, cardiovascular disease; CHAMPS, Cardiovascular Health in Anxiety or Mood Problems Study; EUC, enhanced usual care; GAD-7, generalized anxiety disorder-7; MINI, MINI International Neuropsychiatric Interview; PHQ, Patient Health Questionnaire; UP, unified protocol

This prospective study is a feasibility randomized controlled trial, of parallel design, comparing the effectiveness of the UP versus enhanced usual care. A total of 50 participants will be recruited from the Queen Elizabeth Hospital, a tertiary hospital in the western urban area of Adelaide, South Australia. Parallel to the main trial, a nonrandomized comparator cohort will be recruited comprising at least 150 persons scoring below the predetermined depression and anxiety severity thresholds. Figure 1 depicts the trial design. The trial will be conducted in accordance with the CONSORT statement [50].

### Inclusion criteria

Inclusion criteria are as indicated below:

1. Age ≥ 18 years.
2. A primary hospital admission for CVD (specified by relevant International Classification of Disease codes for coronary artery disease, myocardial infarction, heart failure, atrial fibrillation, other ventricular or atrial arrhythmia, coronary revascularization intervention, symptomatic coronary heart disease including unstable angina pectoris, or heart valve disease).
3. A MINI International Neuropsychiatric Interview (MINI) diagnosis by randomization naïve assessors of major depression, dysthymia, GAD, panic disorder, agoraphobia, social anxiety/phobia, or post-traumatic stress disorder.
4. Above the severity threshold for depression PHQ scores (≥10) [51] or anxiety threshold GAD scores (≥7) [52].
5. Fluency in English.

### Exclusion criteria

Exclusion criteria include the following:

- A psychosis or bipolar disorder diagnosis determined by medical history or randomization-naïve assessors.
- High suicide risk at psychiatric interview.
- Observed cognitive impairment or dementia impeding delivery of psychotherapy or ability to provide informed consent.
- Neurodegenerative condition (for example, Parkinson’s disease or multiple sclerosis)
- In receipt of GP, psychologist or psychiatrist counselling elsewhere.
- A diagnosis of drug and alcohol dependence or abuse determined by randomization-naïve assessors.
- Medical condition likely to be fatal within 1 year.

### Nonrandomized comparator cohort

Participants scoring below the depression and anxiety severity threshold on the PHQ-9 and GAD-7, and therefore those without any distress, will be part of a nonrandomized comparator cohort. The rationale for the comparator cohort is to answer empirical questions about EDBs, incident emotional disorders and trajectories of emotional distress. Several recent RCTs have adopted a similar approach and recruited a comparator cohort for observational purposes.

### Non-randomized comparator cohort eligibility

1. Age ≥ 18 years.
2. A primary hospital admission for CVD (specified by relevant International Classification of Disease codes for coronary artery disease, myocardial infarction, heart failure, atrial fibrillation, other ventricular or atrial arrhythmia, coronary revascularization intervention, symptomatic coronary heart disease including unstable angina pectoris, or heart valve disease).
3. Free from any MINI diagnosis by randomization-naïve assessors of major depression, dysthymia, GAD, panic disorder, agoraphobia, social anxiety/phobia, and post-traumatic stress disorder.
4. Free from any MINI diagnosis by randomization-naïve assessors or medical history of psychosis or bipolar disorder.
5. Below the severity threshold for depression PHQ scores (≤ 9) [51] or anxiety threshold GAD scores (≤ 6) [52].
6. Fluency in English.

### Proposed sample

The proposed sample for this study was 50 patients randomized to the UP or EUC arm. Parallel to the RCT, 150 persons will be recruited into the nonrandomized comparator cohort. In the total sample (N = 200), incorporation of the nonrandomized comparator cohort (n = 150) and RCT participants (n = 50) will enable us to explore the rates of EDBs, physical activity, medication adherence, hospital readmission and feasibility of a screen-and-treat model of care.

### Planned statistical analysis

Data management is outlined elsewhere in our protocol. Comparisons between UP, EUC and the nonrandomized comparator cohort will be made at each follow-up time point on patient level of satisfaction with aspects of psychosocial care. We will evaluate whether screening, education, and support in EUC is a suitable alternative to UP. We will also monitor linkages with primary care to determine whether our suicide risk management strategy is viable when routine depression screening is implemented. These data will help inform the design and day-to-day running of a larger more definitive RCT. In terms of feasibility, we will also evaluate the recruitment eligibility, acceptance, and attrition rates, the length of time taken to recruit, and compliance with the psychosocial questionnaire battery.

In the total sample, incorporating the nonrandomized comparator cohort, the association between EDBs, physical activity, and medication adherence with readmission will be examined. Incident psychological distress and distress trajectories will be evaluated in multi-level models to provide a snapshot of different distress trajectories on each of the measures used in CHAMPS. The longitudinal changes in QOL will also be analyzed in the same manner as incident psychological distress and distress trajectories described above.

### Randomization

An independent statistician will generate the randomization codes. Patients will be block randomized according to a random number generator in alternating block sizes of three and six. Allocation will be concealed in sequentially numbered, opaque, sealed envelopes. Randomization will be stratified by primary CVD admission (myocardial infarction, heart failure, atrial fibrillation, other ventricular or atrial arrhythmia, coronary revascularization intervention, symptomatic CHD including angina pectoris, and heart valve disease) to obtain approximately equal sample sizes in both groups.

### Transdiagnostic unified protocol intervention

The UP is a transdiagnostic cognitive-behavioral intervention explicitly designed to address the full range of emotional disorders (anxiety, depressive, and related disorders); this work is done by targeting core, underlying emotional processes that lead to the development and maintenance of symptoms across disorders. The UP is designed for weekly and face-to-face delivery over 12 to 18 sessions. The UP consists of eight modules: (1) enhancing motivation for change and treatment engagement, (2) facilitating better understanding of patients’ emotional experiences, (3) increasing present focused emotion awareness, (4) increasing cognitive flexibility, (5) identifying and preventing patterns of emotion avoidance and maladaptive EDBs, (6) increasing awareness and tolerance of emotion-related physical sensations, and (7) interoceptive and situation-based emotion-focused exposure, and (8) the final module is devoted to summarizing the relevant techniques attained and to developing relapse prevention strategies. A summary of the UP decision tree and intervention outline is shown in Table 1. A more complete description by Barlow et al. [53] can be found elsewhere regarding the development of the UP and long-term treatment outcomes. An experienced psychologist, who is trained in the UP and trained by the research team on CVDs, will deliver the intervention. In psychotherapy intervention trials, blinding of the study participants, therapists, and study coordinator is not possible. However, all other members of the research team will remain blinded. Intervention fidelity will be maintained by weekly supervision and monitored to determine if the UP requires modification in a larger trial among CVD patients.

**Table 1:** A Description of the eight modules for the transdiagnostic unified protocol in the intervention group

		Transdiagnostic unified protocol
Module	Session	Content
1. Preliminary module	1	Focusing on enhancing motivation and readiness for change and treatment engagement
2. Psychoeducation	2	Educating patients on the nature of emotions and providing a framework for understanding their emotional experiences
3. Present focused awareness	3 – 4	Increasing present focused emotion awareness
4. Cognitive flexibility	5 – 7	Increasing cognitive flexibility
5. Emotion-driven behaviors	8 – 11	Identifying and preventing patterns of emotion avoidance and maladaptive emotion-driven behaviors
6. Emotion awareness and tolerance	12 - 15	Increasing awareness and tolerance of emotion-related physical sensations
7. Exposure	16 – 17	Interoceptive and situation-based emotion focused exposure
8. Summary	18 (or earlier if required)	Summarizing the relevant techniques attained and developing relapse prevention strategies.

### Enhanced usual care

Patients randomized to the EUC group will receive an education package delivered by the study coordinator consisting of the beyondblue™ fact sheet regarding anxiety, depression, and coronary heart disease [54]. Participants and their general physician will be informed of the baseline distress results and directed to available clinical services (psychologist or psychiatrist), advising participants to seek assistance for achieving mental wellbeing with the support of their general physician. This conforms to the National Heart Foundation of Australia’s™ guidelines [51, 55]. There are no restrictions on usual care.

### Standard of care

All participants will receive standard medical care for CVDs according to international guidelines regardless of the study’s allocation. Standard CVD care includes referral to cardiac rehabilitation, which includes a structured exercise regime, and education about CVD risk factors such as stress, dietary modifications and tobacco smoking cessation. All study participants are permitted to seek psychopharmacology, which will be monitored, from outside the study.

### Procedure

The potentially eligible patients are being identified at the hospital 1 to 4 days after their CVD admission by an authorized hospital staff member employed as a trial coordinator in the cardiology department. A pool of eligible participants will be determined by the trial coordinator’s review of cardiology admissions and medical records if required. In the first instance, persons will be approached on the hospital ward by the trial coordinator and provided with the study information sheet and an opportunity to discuss any aspects of the study with the trial coordinator. The potentially eligible patients are re-contacted by the study’s trial coordinator by letter and then telephone 2 to 8 weeks after their CVD admission. Eligible and consenting participants will provide written informed consent at the baseline appointment. Determination of the depression and anxiety thresholds will be made at baseline. Randomization and patient allocation to trial arms will take place by means of a random number generator prepared in advance by an independent statistician offsite. All participants undergo active monitoring for CVD events and mental well-being (PHQ-9 and GAD-7) through a scheduled phone call every 4 to 6 weeks after randomization. The numbers of eligible and successfully recruited patients will be monitored and discussed on a weekly basis between the CI and trial manager.

### Patient rated measures

Psychosocial outcomes will be assessed with a battery of fully validated measures at baseline, after the intervention completion (12 to 18 weeks) and during the 6-month follow-up period. The timing of assessments is shown in Table 2. The self-rated measures will be collected to provide the standard deviation of patient outcomes, which is necessary to perform a power calculation in a larger more definitive trial. Patient rates of alcohol and tobacco use, physical activity, and hospital readmissions will be collected to provide an estimate for a larger more definitive trial.

**Table 2:** Assessment schedule for participants through the study

			Timing of assessment for all participants					
		Eligibility	Pre-random ization	4 weeks	8 weeks	12 weeks	18 weeks	6 months
Variable	Measure							
Inclusion criteria								
Depression	PHQ-9	✓		✓	✓	✓	✓	✓
Generalized anxiety	GAD-7	✓		✓	✓	✓	✓	✓
Psychiatric diagnosis	MINI	✓					✓	✓
Post-eligibility								
Anxiety severity	OASIS		✓				✓	✓
General stress	DASS-21		✓				✓	✓
QOL	SF-12		✓				✓	✓
CVD outcome	MACE						✓	✓
Physical activity	Exercise		✓				✓	✓
GATS	Tobacco		✓				✓	✓
AUDIT-C	Alcohol		✓				✓	✓
MOS SAS	Adherence		✓					
Psychiatric service, medication usage, satisfaction with care	Self-report, audit		✓				✓	✓

AUDIT-C, Alcohol Use Disorders Identification Test-Shortened Clinical Version; CVD, cardiovascular disease; DASS-21, Depression, Anxiety Stress Scales; GAD-7, Generalized Anxiety Disorder-7; GATS, Global Adult Tobacco Survey; MACE, major adverse cardiac event; MINI, MINI International Neuropsychiatric Interview; MOS SAS, Medical Outcomes Study Specific Adherence Scale; OASIS, Overall Anxiety Severity And Impairment Scale; PHQ-9, Patient Health Questionnaire-9; SF-12, Medical Outcomes Study Short Form-12; QOL, quality of life;

### Anxiety, depression and stress symptoms

Generalized anxiety symptoms will be measured by the 7-item Generalized Anxiety Disorder scale (GAD-7). The GAD-7 severity threshold for clinically relevant symptoms is a total GAD score ≥ 7 [52]. This questionnaire has favorable validity as a tool to identify depression and anxiety disorders in medical patients [52]. The Overall Anxiety and Severity Impairment Scale (OASIS) is a five-item brief measure of anxiety symptoms, avoidance, and severity used in a number of large RCTs [56]. Scores > 8 are indicative of severe anxiety. Depression symptoms will be measured by the 9-item Patient Health Questionnaire (PHQ-9). The PHQ-9 severity threshold for clinically relevant symptoms is a total PHQ score ≥ 10 [52]. Stress will be measured by the Depression, Anxiety and Stress Scales, a 21-item clinical measure commonly used in Australia, validated in adults to age 90 years [57, 58], and in previous studies in the cardiac surgery population [59, 60]. Mild distress for the Stress scale is > 8.

### Other rating scales

Quality of life will be assessed with the SF-12. The SF-12 is a commonly utilized and generalizable measure of QOL in CVD populations [61, 62]. Other behavioral factors such as smoking and alcohol use are pertinent to cardiovascular functioning and are likely to be EDBs targeted by the UP intervention. Lifetime and current tobacco use will be measured by items from the Global Adult Tobacco Survey [63]. Recent alcohol use will be measured by the Alcohol Use Disorders Identification Test-Shortened Clinical Version, which provides favorable sensitivity and specificity for the detection of problematic drinking [64]. The physical activity questions from the Australian National Health Surveys are used to classify participants as sedentary or having low, moderate, or high levels of physical activity calculated with metabolic equivalents [65].

### Psychiatric disorders

The MINI is a brief structured interview that will be performed by allocation-naïve assessors to determine the primary psychiatric diagnosis and remission at the end of the study [66, 67]. Assessment takes 20 to 40 minutes to complete and has been validated among cardiac patients [68].

### Medical and demographic data

Demographic, comorbidity and CVD condition data will be obtained via project-specific questions. A statewide hospital registry will be used to identify CVD and psychiatric hospital admissions at 6-month follow-up. Major CVD events include myocardial infarction, stroke, coronary revascularization, cardiac failure, and arrhythmia determined with relevant International Classification of Disease Criteria Codes I00-I99 [69]. Admissions for primary psychiatric causes include suicide attempt, deliberate self-harm, and panic disorder (International Classification of Disease Criteria Codes F00-F99) [69]. Electronic data linkage will be used to determine admissions, and adjudication of study outcomes will be performed by an independent panel of cardiologists, blinded to the randomization arm. Patient confidentiality will be maintained.

### Intervention acceptability and feasibility

Participants will be asked to rate how effective different mental health care treatments were. The questions will assess psychologist treatments (a component of CHAMPS) and outside treatments: specifically, psychiatrist, GP counselling, anti-depressant, anti-anxiety medications, and other areas of support.

### Stopping rules

If there are concerns about significant emerging psychiatric symptoms (for example, psychosis, mania or other serious mental health conditions) associated with risks to the participant or others, and particularly if the PHQ score is ≥ 20 or the PHQ-9 item 9 score ≥ 2, the Mental Health Triage will necessarily be contacted, or alternatively, referral to an emergency department will be undertaken if a risk is imminent and high. If significant symptoms or comorbidities (for example, psychosis, hypomania, or substance use) that do not meet the previous criteria emerge at any point during the study, the psychologist will stop the study protocol, treat these conditions and/or provide appropriate referral for psychiatric or drug and alcohol service review. Psychiatric referral should be considered if there are significant risk factors for self-harm or CBT resistance such as personality disorder, past self-harm attempts or treatment-resistant depression.

### Ethical considerations

The study has been approved by Human Research Ethics Committee of The Queen Elizabeth Hospital (approval #HREC/15/TQEH47). Written informed consent will be obtained from the participants before inclusion. Study participants are free to withdraw from the study at any time. The study carries a suicide risk, and therefore, the patients’ levels of suicidal ideation will be monitored with the PHQ-9 (every 4 weeks). As in our real-world clinic, we will follow a protocol for managing suicidal intent and self-harm attempts [38]. An independent data monitoring board will monitor adverse events (cardiac, psychiatric, and other) and report these to the governing ethics board. No plans are made to audit the conduct of the trial until it is completed.

## Discussion

The proposed feasibility RCT intervention for emotional disorders will provide an innovative treatment that potentially better aligns with the complex mental health needs for persons afflicted with CVDs. This is perhaps the first investigation of its kind to comprehensively treat a broad range of emotional disorders in CVDs utilizing a single set of therapeutic principles. If the UP is feasible and acceptable, we will develop a larger more definitive trial, and therefore, the findings may hold relevance for the design of future trials, healthcare service delivery, and funding in cardiology.

Several limitations of this trial exist, including that it is applied to multiple emotional disorders, and potential heterogeneity will exist in the study participants’ treatment needs. In addition, the findings will require replication, perhaps in larger numbers or more homogenous samples.

At the feasibility stage of intervention development, the establishment of superiority of the UP from TAU is important. Definitive identification of the specific components of the active treatment that fosters change in anxiety, depression, and quality of life among CVD patients will not be possible. Moreover, the length of follow-up should be extended in a larger trial to determine the effects on CVD events.

The present feasibility RCT is designed to evaluate the UP targeting emotional disorder processes in a CVD population, with particular focus on acceptability, acceptance rates, satisfaction with care and attrition. If the trial is viable, it opens up the possibility for interventions to target broader emotional processes in the precarious population with CVD and emotional distress and paves the way for a larger more definitive trial. With increasing recognition of the role of negative emotions in CVDs, the need for depression disorder-only treatments could become increasingly obsolete in CVDs as developments in treatments match those in clinical psychology [46].

## Trial status

The trial has been approved and is currently recruiting.

## Abbreviations

CVD: cardiovascular disease
CHAMPS: Cardiovascular Health in Anxiety or Mood Problems Study
EDB: emotion-driven behavior
EUC: enhanced usual care
GAD: generalized anxiety disorder
MACE: major adverse cardiac event
MINI: MINI International Neuropsychiatric Interview
OASIS: Overall Anxiety Severity And Impairment Scale
PHQ: Patient Health Questionnaire
QOL: quality of life
UP: unified protocol
